# Intravenous Lidocaine and Ketamine Infusions for Headache Disorders: A Retrospective Cohort Study

**DOI:** 10.3389/fneur.2022.842082

**Published:** 2022-03-09

**Authors:** Jason C. Ray, Shuli Cheng, Kirsten Tsan, Hassan Hussain, Richard J. Stark, Manjit S. Matharu, Elspeth Hutton

**Affiliations:** ^1^Department of Neurology, Austin Health, Melbourne, VIC, Australia; ^2^Department of Neurology, Alfred Hospital, Melbourne, VIC, Australia; ^3^Department of Neuroscience, Monash University, Melbourne, VIC, Australia; ^4^School of Medicine, Nursing and Health Sciences, Monash University, Melbourne, VIC, Australia; ^5^Headache and Facial Pain Group, University College London Queen Square Institute of Neurology, National Hospital for Neurology and Neurosurgery, London, United Kingdom; ^6^Headache and Facial Pain Group, University College London, London, United Kingdom

**Keywords:** migraine, headache disorders, lidocaine, ketamine, status migrainosus, medication overuse headache

## Abstract

**Introduction:**

The use of lidocaine (lignocaine) and ketamine infusion in the inpatient treatment of patients with headache disorders is supported by small case series. We undertook a retrospective cohort study in order to assess the efficacy, duration and safety of lidocaine and ketamine infusions.

**Methods:**

Patients admitted between 01/01/2018 and 31/07/2021 were identified by ICD code and electronic prescription. Efficacy of infusion was determined by reduction in visual analog score (VAS), and patient demographics were collected from review of the hospital electronic medical record.

**Results:**

Through the study period, 83 infusions (50 lidocaine, 33 ketamine) were initiated for a headache disorder (77 migraine, three NDPH, two SUNCT, one cluster headache). In migraine, lidocaine infusion achieved a ≥50% reduction in pain in 51.1% over a mean 6.2 days (SD 2.4). Ketamine infusion was associated with a ≥50% reduction in pain in 34.4% over a mean 5.1 days (SD 1.5). Side effects were observed in 32 and 42.4% respectively. Infusion for medication overuse headache (MOH) led to successful withdrawal of analgesia in 61.1% of lidocaine, and 41.7% of ketamine infusions.

**Conclusion:**

Lidocaine and ketamine infusions are an efficacious inpatient treatment for headache disorders, however associated with prolonged length-of-stay and possible side-effects.

## Introduction

Within Australia, headache was the 20th most common diagnosis for patients subsequently admitted to hospital for the period 2016–2017 (1). Accordingly, headache is associated with a significant economic impact, with an estimated $6.8 billion in 2018 in hospital admissions and emergency department presentations (1). First-line therapies for patients presenting with a primary headache disorder to the emergency department, and in particular migraine, is supported by moderate or high-quality evidence (2).

In patients who fail to respond to initial therapies such as non-steroidal anti-inflammatory drugs, triptans, prochlorperazine or chlorpromazine, there are limited treatment options with a high-level of evidence (2). In a recent survey of American Headache Society (AHS) members, intravenous lidocaine (lignocaine) was recommended as a first line therapy for hospitalized migraine patients in 4.3% and second line therapy in 6.5% of respondents, while intravenous ketamine was recommended in 1.1 and 4.3% respectively (3).

The use of continuous intravenous lidocaine as an acute abortive treatment in migraine or chronic daily headache is supported by several case series (4–7), as is the use of intravenous ketamine (8). Brief one hour infusions of lidocaine has also recently been studied in a controlled trial as a treatment in the emergency department (9). The use of intravenous lidocaine and ketamine require prolonged hospital admission, and are associated with potential adverse effects including neuro-psychiatric and cardiac side effects (5, 10). Within our institution, patients have been referred by treating clinicians for lidocaine and ketamine infusions in treatment of several headache disorders both as an acute abortive therapy, and as a semi-elective procedure in treatment of refractory disease or medication overuse. Lidocaine or ketamine was considered where the patient had failed to respond to appropriate first-line treatments of the condition such as triptans, simple analgesia and prochlorperazine. In this cohort study we sought to assess the efficacy, duration and safety of lidocaine and ketamine infusions at our institution.

## Methods

A retrospective cohort study was undertaken of patients treated with intravenous lidocaine or ketamine while admitted under the neurology unit of a single Australian tertiary hospital due to a headache disorder. To ensure accurate data, cases were identified from both ICD coding and prescription of intravenous lidocaine or ketamine on the hospital electronic prescription system between 1st January 2018 and 31st July 2021. Local protocols for management of lidocaine and ketamine infusions are included as attachment one and two respectively (Supplementary Materials). Cases were reviewed by a specialist neurologist to ensure accuracy of documentation and data was then extracted from the electronic medical record (EMR).

We primarily examined the reduction in visual analog scale (VAS) pain score, which was determined by analysis of recorded pain score in the medical record at the commencement and cessation of infusion (or admission and discharge). Demographic data, medical history, length of stay, infusion, complication and re-presentation were all recorded from the EMR. The number of previously failed preventative medications for migraine were recorded where available from treating specialist correspondence and hospital clinic letters (data available in 47/77 migraine patients). Headache diagnoses were made according to the International Classification of Headache Disorders (ICHD-3).

A variance and regression analysis were undertaken to assess adjusted outcomes in the cohort, and raw outcome data was also reported. *T*-tests used to test the null hypothesis. Two-sided *p*-values <0.05 were considered statistically significant. All analyses were performed in SPSS v27.0. This study received approval from the local human research ethics committee (HREC 327/20).

## Results

Between 01 January 2018 and 31 July 2021 eighty-three patients were admitted to hospital for the treatment of a headache disorder and required an intravenous infusion of either lidocaine or ketamine. The prescription and management of intravenous infusions at the hospital is standardized and managed by local protocols. Through the study period, fifty patients received lidocaine infusions at a rate of 2 mg/min. A total of 33 patients received ketamine infusions, which are started at 7 mg/h, and up-titrated according to protocol (see attachment 2) and as tolerated to a maximum dose to 24 mg/h.

A total of 77 patients received an infusion for migraine (45 lidocaine, 32 ketamine), three for new daily persistent headache (NDPH) (two lidocaine, one ketamine), two patients received lidocaine infusions for SUNCT and one received a lidocaine infusion for cluster headache. The demographics of the cohort are presented in Table 1. There were significant differences between the patient cohorts in the mean number of comorbidities and mean number of previously failed preventative medications, with higher means seen in the ketamine cohort. Lidocaine infusions were associated with a significantly longer infusion.

**Table 1:** Baseline characteristics of patient cohort.

	**Cohort *N* = 83**	**Lidocaine *N* = 50**	**Ketamine *N* = 33**	
Mean age (SD)	42.6 (15.2)	42.9 (16.2)	42.2 (13.9)	*P* = 0.848
Female n (%)	69 (83.1%)	39 (78%)	30 (90.9%)	
Migraine	77 (92.8%)	45 (90%)	32 (97%)	
NDPH	3 (3.6%)	2 (4%)	1 (3%)	
SUNCT	2 (2.4%)	2 (4%)	-	
Cluster Headache	1 (1.2%)	1 (2%)	-	
Mean comorbidities (SD)	3.3 (2.6)	2.8 (2.5)	4.1 (2.9)	*p* = 0.024
Mean failed preventers (SD)	7 (4.2)	5.8 (3.8)	8.7 (4.1)	*p* = 0.013
Mean duration infusion (days, SD)	5.8 (2.1)	6.2 (2.4)	5.1 (1.5)	*p* = 0.021

*NDPH, new daily persistent headache; SUNCT, short-lasting unilateral neuralgiform headache attack with conjunctival injection and tearing*.

The overall rate of any adverse effect of intravenous infusion was 32% in the lidocaine cohort, and 42.4% in the ketamine cohort. The majority of adverse events reported were minor, and not unexpected with the medication, such as paraesthesia with ketamine infusion and dizziness with lidocaine, however more significant adverse effects were noted requiring cessation of infusion. A complete list of adverse events is summarized in Table 2. In total, 10% (5/50) of lidocaine infusions had an adverse event that led to cessation, and 2% (1/50) continued following dose reduction. A total of 12.1% (4/33) of patients who received a ketamine infusion had an adverse event that led to cessation, while 3% (1/33) continued after dose reduction.

**Table 2:** Side-effects of lidocaine and ketamine infusion.

	**Lidocaine *N* = 50**	**Ketamine *N* = 33**
Anxiety or agitation	4 (8%)	1 (3%)
Dizziness or light headed	3 (6%)	1 (3%)
Arrhythmia	2 (4%)	-
Hypertension	-	1 (3%)
Hypotension	3 (6%)	-
ACS or confusion	1 (2%)	2 (6.1%)
Chest pain	1 (2%)	-
Paraesthesia	1 (2%)	4 (12.1%)
Rash	1 (2%)	-
Nausea	1 (2%)	-
Hallucination	-	2 (6.1%)
PNES	-	1 (3%)
Weakness	-	1 (3%)

*PNES, psychogenic non-epileptiform seizure; ACS, altered conscious state*.

### Migraine

A total of 77 patients received infusions for migraine (45 lidocaine, 32 ketamine) over the study period. Of the cohort, 68.9% were semi-electively admitted to the hospital for the purposes of the infusion, while the remainder received an infusion after presenting through the emergency department and an escalation of other therapies. Medication overuse in this cohort was present in 39%, and 33.8% of patients presented in status migrainosus. The mean maximum tolerated dose of ketamine was 16.3 mg/h (SD 6.5 mg). The demographics of the migraine cohort are summarized in Table 3.

**Table 3:** Characteristics of migraine patients.

	**Cohort *N* = 77**	**Lidocaine *N* = 45**	**Ketamine *N* = 32**	
Mean age (SD)	42 (14.9)	42.4 (15.8)	41.6 (13.5)	*p* = 0.814
Female n (%)	67 (87%)	37 (82.2)	30 (93.8)	
Chronic migraine n (%)	70 (90.9%)	39 (86.7%)	31 (96.9%)	
Elective infusions n (%)	53 (68.8%)	28 (62.2%)	25 (78.1%)	
Status migrainosus n (%)	26 (33.8%)	16 (35.6%)	10 (31.3%)	
MOH n (%)	30 (39.0%)	18 (40%)	12 (37.5%)	
Mean comorbidities (SD)	3.4 (2.6)	2.9 (2.3)	4.1 (2.9)	*p* = 0.049
Mean failed preventive treatments (SD)	7.5 (4.1)	6.4 (3.8)	8.9 (4.1)	*p* = 0.037
Mean Duration infusion (days, SD)	5.8 (2.1)	6.2 (2.4)	5.1 (1.5)	*p* = 0.017

*MOH, medication overuse headache*.

In patients who received a lidocaine infusion, the proportion who achieved a 50% or greater reduction in pain was 51.1% (23/45), achieved after a mean of 4.5 (SD 0.5 days), while 31.1% (14/45) achieved pain freedom over a mean infusion duration of 6.2 days (SD 2.4 days). The 30-day re-admission rate was 6.7% (3/45). In the ketamine infusion cohort, the proportion of patients who achieved a 50% or greater reduction in pain was 34.4% (11/32), achieved after a mean of 3.4 days (SD 0.5 days) while 15.6% (5/32) achieved pain freedom over a mean of 5.1 days (SD 1.5). The 30-day re-admission rate was 3.1% (1/32).

**Figure 1:**
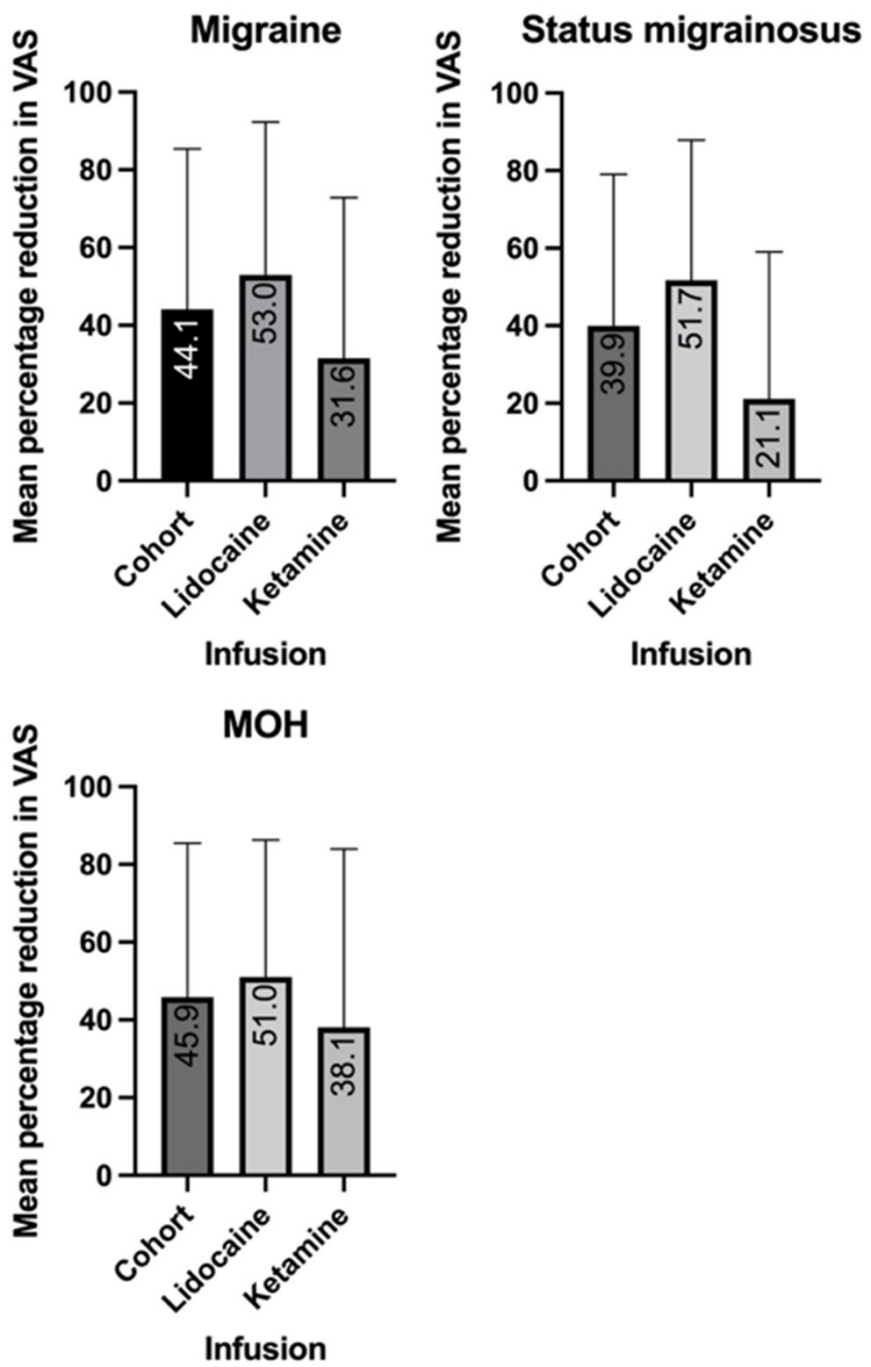
Mean reduction in visual analog scale (VAS) score with intravenous lidocaine and ketamine in migraine, medication overuse headache (MOH) and status migrainosus.

In the migraine cohort, the mean percentage reduction in VAS pain score with lidocaine infusion was 53% (SD 39.3), and 31.6% (SD 41.3) for ketamine infusion (Figure 1). In 47 of the 77 cases data was available for previously trialed preventative medications, and these patients were included in a univariate analysis of variance. The unadjusted mean reduction in VAS pain score in this smaller group was 62.6% (SD 38.8) for lidocaine and 32.7% (SD 42.6) for ketamine. A univariate analysis of variance was undertaken to explore the mean reduction in VAS between the cohorts, controlling for the number of comorbidities and failed preventive treatments as co-variates. This revealed a statistically significant difference between the infusion groups (*p* = 0.035). The estimated marginal mean percentage reduction following co-variate analysis was 62% in the lidocaine group (95% CI 45–79%), and 33.4% in the ketamine group (95% CI 14.4–52.4%).

### Complications of Migraine

Within the migraine cohort 26 patients were reported to suffer from status migrainosus. Within this group, the 16 patients received a lidocaine infusion, 43.8% (7/16) of whom had a ≥50% reduction in pain, while 25% (4/16) achieved pain freedom over a mean 6.6-day (SD 2.7) infusion. The 30-day re-admission rate was 12.5% (2/16). A total of 10 patients received a ketamine infusion, with a ≥50% reduction in pain rate of 20% (2/10) and pain freedom in 10% (1/10), over a mean 5.9 day (SD 1.2). There were no re-admissions within 30 days.

Within the migraine cohort, 30 patients were documented to suffer from medication overuse headache (MOH). Of patients who received a lidocaine infusion, 27.8% (5/18) overused triptans, 5.5% (1/18) overused NSAIDs and 72.2% (13/18) overused opiates. Within the ketamine cohort, 16.7% (2/12) overused triptans, 16.7% (2/12) overused NSAIDS and 66.7% overused opiates. Within the MOH cohort, lidocaine infusion achieved a ≥50% reduction in pain in 50% (9/18) of patients, and pain freedom in 22.2% (4/18). The offending was successfully withdrawn in 61.1% (11/18), and the mean duration of infusion was 6.1 days (SD 2.3). Ketamine infusion achieved a ≥50% reduction in pain in 25% (3/12), and pain freedom in 25% (3/25). Medication overuse was successfully addressed in 41.7% (5/12) patients, over a mean duration of 5.3 days (SD 1.3).

### New Daily Persistent Headache

Three patients received infusions (two lidocaine, one ketamine) for a new daily persistent headache (NDPH). Both patients who received lidocaine infusion (one elective, one via emergency) achieved a ≥50% reduction in pain, while neither had pain freedom. The patient who received a ketamine infusion electively did not achieve a ≥50% reduction in pain. No patients were re-admitted within 30 days.

### Trigeminal Autonomic Cephalalgia

Within the study period, three patients received a lidocaine infusion for a trigeminal autonomic cephalalgia (TAC), two patients diagnosed with SUNCT (one elective, one via emergency) and one with cluster headache (via emergency). All patients who received a lidocaine infusion for a TAC achieved both a ≥50% reduction in pain and pain freedom. One patient with SUNCT re-presented within 30 days.

## Discussion

This study provides further evidence for the use of intravenous lidocaine and ketamine in the treatment of headache disorders. The efficacy of intravenous lidocaine in achieving pain freedom at discharge in status migrainosus in this cohort (25%) is similar to the 20% reported previously in a child and adolescent cohort (5). A smaller adult series previously reported pain freedom in 4/5 infusions (4). In treatment of MOH, the rates of analgesic withdrawal was lower in our cohort (61.1 vs. 97%) and pain freedom (22.2 vs. ~60%) then those previously reported by Williams and Stark (6). Direct comparisons between previously published series and our cohort study are fraught however due to both methodological differences between the studies, and the high number of preventative treatment failures in our group, suggesting a more refractory cohort.

The efficacy of ketamine infusions has been reported previously by Lauritsen et al. In this case series, the authors utilized a VAS < 3 as an outcome measure rather than pain freedom, which accounted for at least a ≥50% reduction from the reported initial VAS. The authors reported 6/6 patients achieved this outcome. In comparison in our cohort, the ≥50% reduction rate in migraine was significantly less (34.4%) (8).

The reported rate of adverse-effects of intravenous lidocaine in the literature varies between 0 and 100% (10). The rate of psychiatric and cardiac adverse events in our cohort was lower than that reported by Gil-Gouveia and Goadsby (50% each respectively) (10), while the overall adverse event rate was higher than the largest previous lidocaine case series (6). Conversely, Hand et al. reported adverse events in 4/19 cases (4). Overall, the adverse effects of intravenous lidocaine in our cohort were generally mild and transient, with a discontinuation rate for any reason of ~10% in each cohort.

The mechanism by which intravenous lidocaine has anti-nociceptive effect in headache is uncertain. It has an inhibitory effect on voltage-gated sodium channels, and the active metabolite of lidocaine, monoethylglycine (MEG) inhibits the glycine transporter GlyT1 (11). In pre-clinical models, GlyT1 inhibition has been shown to reduce allodynia, as well as normalize neuronal, voltage-gated sodium channels and c-fiber firing (12). There is evidence also that lidocaine attenuates IL-6, which has a role in hyperalgesia and allodynia (12). The mechanism by which ketamine may have anti-nociceptive effect in headache is postulated to relate to its role as a NMDA receptor antagonist. Inhibition of NMDA in turn inhibits glutamate, which has been implicated in cortical spreading depression propagation, central sensitization and activation of nociceptive neurons (8). Other parenteral treatment options include dihydroergotamine, which inhibits vasoconstriction, and CGRP release at 5HT1B/D receptors (13), chlorpromazine which antagonizes dopamine (14) and sodium valproate, which inhibits GABA uptake and inhibits voltage-sensitive channels while stimulating GABA synthesis (15).

Consideration of the preventative medication that a patient has responded to may provide insight into the tolerability of the infusion. Propranolol and verapamil, which may cause bradycardia, may be poorly tolerated with lidocaine. Ketamine undergoes N-demethylation in the liver under the influence of CYP3A4 (16), while lidocaine undergoes N-demethylation under the influence of CYP1A2 and CYP3A4 (17). Inhibitors of CYP3A4 include verapamil, while carbamazepine is an inducer of both CYP3A4 and CYP1A2 (18, 19).

The strengths of this study include the size of the cohort, which is the largest infusion study in the literature that the authors are aware of. In addition, outcome data was available for every case, and each case was reviewed by a specialist neurologist. The major limitation of this study is the retrospective design. Data such as the presence of complications of migraine such as status migrainosus or MOH relied upon the accuracy of documentation at the time, and as such are most liable to under-reporting in this study. Data detailing all trialed alternate therapies, timing of treatment failure, and duration of symptoms prior to commencement of infusion was not available. Similarly, data following discharge was not available, and as such duration of effect, MOH withdrawal or pain freedom is not known. Finally, the choice of intravenous infusion for individual patient was made by the treating neurologist at the time, and therefore is subject to patient factors, as well as individual prescribing habits and biases.

## Conclusion

This cohort study provides further evidence for the efficacy of intravenous lidocaine and ketamine in the inpatient treatment of headache disorders. Our study suggests that lidocaine infusions are associated with a higher mean reduction in pain score compared to ketamine, however further prospective study is required to confirm this observation. The overall adverse effects of both infusions in this cohort was high. Further prospective, controlled studies are required to guide treatment decisions in the inpatient treatment of migraine and headache disorders.

## Data Availability Statement

The raw data supporting the conclusions of this article will be made available by the authors, without undue reservation.

## Ethics Statement

The studies involving human participants were reviewed and approved by Alfred Health Human Research Ethics Committee. Written informed consent for participation was not required for this study in accordance with the national legislation and the institutional requirements.

## Author Contributions

JR and EH developed the concept of the project. JR, KT, HH, and SC contributed to data extraction. JR was responsible for the primary writing of the manuscript. All authors contributed substantially to data analysis, editing, and approval of the final manuscript.

## Conflict of Interest

JR has received payment for educational presentations from Viatris. RS has served on advisory boards for Novartis, Teva, Eli Lilly, Allergan, Lundbeck and has received payment for educational presentations from Allergan, Teva, Eli Lilly and Novartis. MM serves on the advisory board for Allergan, Novartis, Eli Lilly, Autonomic Technologies Inc. and TEVA and has received payment for the development of educational presentations from Allergan, electroCore, Eli Lilly, Novartis and TEVA. EH has served on advisory boards for Sanofi-Genzyme, Novartis, Teva, Eli Lilly, Allergan, Lundbeck, been involved in clinical trials sponsored by Novartis, Teva, Xalud, Cerecin, and has received payment for educational presentations from Allergan, Teva, Eli Lilly and Novartis. The remaining authors declare that the research was conducted in the absence of any commercial or financial relationships that could be construed as a potential conflict of interest.

## Publisher's Note

All claims expressed in this article are solely those of the authors and do not necessarily represent those of their affiliated organizations, or those of the publisher, the editors and the reviewers. Any product that may be evaluated in this article, or claim that may be made by its manufacturer, is not guaranteed or endorsed by the publisher.
