# Supplementary material for: Intravenous Lidocaine and Ketamine Infusions for Headache Disorders: A Retrospective Cohort Study
Source: Front Neurol. 2022 Mar 9;13:842082. doi: 10.3389/fneur.2022.842082 (PMC8959588; doi:10.3389/fneur.2022.842082)
Supplement: Supplementary file 1 [file Data_Sheet_1.PDF]

Title **LIGNOCAINE INFUSION FOR NEUROPATHIC PAIN**

This drug guideline has been prepared to standardise the prescribing, administration and dispensing of this specific medication. Additional information relating to this drug can be found in the references listed and by contacting the Alfred Medicines Information Service on 62002.

Areas Applicable: Alfred– All Areas under the direction of Palliative Care and Acute Pain Services

**Description**	
**Drug Presentation**	• Lignocaine 500 mg in 5 mL (**Xylocard® 10%**)<br>• **Note: Some lignocaine products also contain adrenaline, check carefully that the correct strength & product has been selected**
**Prescribing Requirements /Restrictions**	• Use for neuropathic pain treatment or pain relief in burns must be initiated by Acute Pain Services (APS) or Palliative Care Unit.
**Drug Storage/ Availability**	• Store ampoules below 25 degrees C.
**Action (Pharmacology)**	Lignocaine is an amide local anaesthetic which produces analgesia by blockade of peripheral and central sodium ion channels. The prevention of sodium influx will inhibit propagation of action potential to block pain conduction by nerves. Lignocaine has been used for the following indications:<br>• Neuropathic pain.<br>• Chronic daily headache in patients with rebound headache / migraine (see Lignocaine infusion for analgesic rebound headache/migraine)<br>• Class 1B cardiac anti-arrhythmic agent for serious ventricular arrhythmias (see 'Lignocaine Infusion for Arrhythmias')<br>• Half- life for infusion is 1.5 to 2 hours; this will be extended in patients with hepatic and/or heart failure. During continuous infusion, steady state is reached after 6 to 8 hours
**Indications**	To be prescribed by either Acute Pain Services or Palliative Care Unit for the treatment of:<br>• Neuropathic pain when standard therapies do not provide adequate pain relief<br>• Pain relief in burns patients (under the direction of Acute Pain Services) –<br>○ Used perioperatively for the treatment of donor site pain.<br>○ Where anti-neuropathic options have already been optimised.<br>○ Considered for burns patients whose background opioids are $\geq$150mg/day MOE (Morphine Oral Equivalent)<br>• Lignocaine infusion can be commenced ONE hour prior to dressing change and continue for ONE hour post dressing change (see 'ICU Management of Procedural Pain for Intubated Burn Patients')<br>• For treatment of serious arrhythmias – see 'Lignocaine Infusion for Arrhythmia'
**Contraindications**	• Patients with sinoatrial or atrioventricular (especially 2nd or 3rd degree) blocks or intraventricular blocks or other conduction defects on 12-lead ECG (unless pacemaker in situ).<br>*Note: A patient with pre-existing **first** degree heart block may receive lignocaine infusions only after obtaining agreement from the Cardiology Unit.*<br>• Pre-existing hypotension (especially if symptomatic)

The hard copy of this document may be out of date. To ensure you are reading the current version, check the policy and procedure site on the Alfred Health Intranet.

**Contraindications Continued**	• Bradycardia<br>• Supraventricular tachycardia e.g. AF<br>• Cardiac failure<br>• Uncontrolled epilepsy<br>• Known hypersensitivity to amide local anaesthetic agents such as prilocaine & bupivacaine (rare)
**Precautions**	• Possible to induce tachycardia via re-entry mechanism e.g. in Wolff-Parkinson-White Syndrome<br>• The infusion should be ceased immediately if patient experiences serious cardiac or respiratory or neurological side effects. Notify medical staff for an urgent review<br>• Postural orthostatic tachycardia syndrome (POTS) – bed rest may exacerbate this condition. Please discuss with the treating cardiologist prior to admission.<br>• Beware of polypharmacy, particularly with agents which may prolong QTc (see Drug interactions).<br>• Lower dose in elderly patients and in those with (see Dose range)<br>– Body weight < 80kg<br>– Obesity (>120kg)<br>– heart failure<br>– liver failure – consider halving the dose in those with severe impairment and continuous cardiac monitoring<br>– renal failure (renal impairment is unlikely to affect lignocaine clearance if duration is < 24 hrs, however, toxicity may still develop after prolonged use with accumulation of a less active metabolite).
**Administration**	
**Pre-treatment requirements**	A thorough medical assessment/ examination is required before starting infusion (refer to ‘Contraindications’ section)<br>• A baseline 12 lead ECG (Chest burns where a 12 lead ECG is unable to be obtained then a 3 Lead ECG will be adequate)<br>• LFT, FBC, U&E, Calcium, Magnesium, Phosphate.<br>• Baseline vital signs - BP, pulse, respiratory rate and temperature<br><br>**Resuscitation equipment** must be made available prior to administration<br><br>**These may not be required in palliative patients at the discretion of the Palliative Care Unit.**
**Dose Range**	**Usual Dose**<br>• 25 mL/hour = 2 mg /min (unless otherwise specified by the Consultant in charge or meets criteria for a lower dose). The maximum dose is 2.4 mg/min,<br>• Consider lower dose (i.e. 12.5 mL/hour) in the following patients:<br>○ body weight < 80kg<br>○ the elderly > 75 years<br>○ heart failure,<br>○ shock<br>○ liver impairment;<br>○ renal impairment<br>NOTE there is a separate dose regime for subcutaneous infusion see below.

The hard copy of this document may be out of date. To ensure you are reading the current version, check the policy and procedure site on the Alfred Health Intranet.

	**Must first ascertain whether patient requires continuous cardiac monitoring. The decision to initiate prolonged lignocaine infusions if continuous cardiac monitoring is required can be made by the Palliative Care or Acute Pain Service only after obtaining agreement from the Cardiology Unit.**<br><br>Patients who require monitoring will be transferred to a ward with monitored beds.<br><br>Continuous cardiac monitoring is required in:<br>1. Patients with pre-existing first degree heart block prior to lignocaine administration AND the Cardiology Unit has specifically requested continuous cardiac monitoring.<br>(Note*: Lignocaine is contraindicated in patients with* 2nd or 3rd *degree heart blocks*)<br>2. Patients in whom the prescribed lignocaine dose is **>2 mg/min**. However, an exception for the need to have continuous cardiac monitoring can be made for<br>○ patients who weigh >70 kg with no cardiac complications, and<br>○ in whom the lower dose of ≤2 mg/ min has proven to be ineffective.<br><br>In these patients, the Palliative Care or APS Consultant may specifically increase the dose up the maximum allowable dose of 2.4 mg/min without requiring continuous cardiac monitoring<br><br>Check fluid and electrolyte status. **Ensure normal K+ level** before starting treatment as both hypo- or hyperkalaemia can increase risk of arrhythmia.<br><br>**Resuscitation equipment** must be made available prior to administration<br>For patients who do not require continuous cardiac monitoring, refer to "Monitoring".
**Intravenous Infusion**	Infusion Concentration: 2400 mg Lignocaine (= 24 mL of 500 mg/5 mL amp)<br>Make up infusion in: 500 mL bag<br>Compatible Solutions: 0.9% sodium chloride<br>Volume to be removed: Nil<br>Final Concentration: 4.8 mg/mL*<br>Usual Dose 2 mg/min (= 25mL) – see dose range<br>Infusion pump: Continuous infusion pump e,g Carefusion Alaris<br><br>** These concentrations do not take into consideration the additional added volume. Additional volume is only calculated for when it exceeds 10% of the total volume*
**Subcutaneous Infusion via NIKI T34 syringe driver**	The Niki T34 pump: Prescription must be written on a subcutaneous prescription chart MR M67 (see Niki T34 guideline)<br>Infusion Concentration: Commence at 500 mg Lignocaine/24 hours<br>If pain inadequately controlled after 6 hours consider titration in increments of 250-500mg to maximum of 2500mg per 24 hours<br>Make up infusion in: 20mL Luer Lok syringe<br>Compatible Solutions: 0.9% sodium chloride to a total volume of 15mLs<br>**For doses greater than 1500mg (15mLs) the dose should be split and run via two Niki T34 infusion pumps simultaneously**

The hard copy of this document may be out of date. To ensure you are reading the current version, check the policy and procedure site on the Alfred Health Intranet.

Title **LIGNOCAINE INFUSION FOR NEUROPATHIC PAIN**

**Titration Parameters**	• Not titrated - infused as ordered by Acute Pain Services / Palliative Care Unit
**How to wean infusion/dose**	• The lignocaine infusion can be ceased abruptly without titrating down
**Incompatibilities**	• Refer to Australian Injectable Drugs Handbook
**Y-Site Compatibility**	• A secondary infusion/injection should not be delivered through the same line as the lignocaine Infusion, unless in case of emergency, as the patient is at risk of receiving a bolus of lignocaine.
**pH**	• 5 – 7
**Practice Points**	
**Side Effects**	*Adverse effects are dose-related and occur more frequently at infusion rates ≥ 3 mg/min. Signs of CNS toxicity usually precede those of the cardiovascular system*<br>**Central nervous system:**<br>• Light headedness/ dizziness/ drowsiness/ restlessness/ confusion<br>• Nausea & vomiting<br>• Perioral tingling or tongue numbness<br>• Twitching/ tremor/ paraesthesia<br>• Seizure/ convulsion<br>• Visual disturbances/ blurred vision<br>• Speech disturbances (e.g. slurred speech)<br>• Tinnitus<br>• Euphoria/ hallucinations<br>• Reduced level of consciousness, coma<br>• Respiratory arrest<br><br>**Cardiovascular system:**<br>• Hypotension<br>• Bradycardia or arrhythmia<br>• AV heart block (new or worsened) or suppression of SA node<br>• May promote tachycardia via re-entry mechanism<br>• Asystole<br>• May decrease effectiveness of DCR<br>Hypersensitivity reactions – occurs rarely e.g. urticaria, rash and anaphylaxis<br>With suspected toxicity a lignocaine toxicity level should be performed (a red top blood tube can be sourced from Main Recovery) as soon as the symptoms are reported
**Monitoring IV infusion protocol**	*Prior to commencement of infusion*<br>• A baseline 12 lead ECG (Chest burns where a 12 lead ECG is unable to be obtained then a 3 Lead ECG will be adequate)<br>• Baseline vital signs - BP, pulse, respiratory rate and temperature<br><br>*After commencing the infusion:*<br>• BP, heart rate and sedation score:<br>o Every hour for 4 hours, then<br>o Every 2 hours for 4 hours, then<br>o Every 4 hours

The hard copy of this document may be out of date. To ensure you are reading the current version, check the policy and procedure site on the Alfred Health Intranet.

	• <u>Pain Score</u>:<br>o Every hour for 4 hours, then<br>o Every 4hours (omit between 2200 and 0600 if asleep)<br>• <u>Daily 12 lead ECG</u> or 3 lead for QTc assessment<br>• <u>Paraesthesia</u>:<br>o Ask patient to report paraesthesia around mouth immediately<br>o Ask about paraesthesia around mouth every 4 hours for first 24hours after initiating lignocaine or adjusting dose<br>o Once patient has been on stable dose for 24hours monitoring can be reduce to every 6 hours<br>• <u>Toxicity/Signs and symptoms of Overdose</u>:<br>o Check for signs of toxicity at least once per shift Document any neurological side effects (even if absent)<br>o Escalate to medical staff if signs of toxicity present.<br><br>**Infusion should be stopped immediately if serious cardiac or neurological or respiratory side effects are suspected.**<br>**If signs/symptoms of above side effects, escalate to medical staff for an urgent review.**<br><br>**If <u>convulsions occur CALL a MET call</u>** and immediately inform Acute Pain Service **4732**<br><br>**Lignocaine levels must be taken – red top blood tubes sourced from Recovery Unit.**
**Monitoring subcutaneous infusion protocol**	Monitoring requirements in palliative care patients to be at the discretion of the Palliative Care Unit. For all other patients monitoring is as follows;<br><br>*<u>Prior to commencement of infusion</u>*<br>• A baseline 12 lead ECG (or 3 lead rhythm strip for patients unable to establish 12 lead, ie burns)<br>• Baseline vital signs - BP, pulse, respiratory rate and temperature<br><br>*<u>After commencing the infusion</u>*<br>*(Monitoring requirements may be reduced at the discretion of the palliative care team)*<br>• <u>BP, heart rate, sedation score</u>:<br>o Every hour for 4 hours for first 24hours after initiating lignocaine or adjusting dose<br>o Once patient has been on stable dose for 24hours monitoring can be reduced to every 6 hours<br>• <u>Pain Score</u>:<br>o Every 6 hours (omit between 2200 and 0600 if asleep)<br>• <u>Paraesthesia</u>:<br>o Ask patient to report paraesthesia around mouth immediately<br>o Ask about paraesthesia around mouth every 4 hours for first 24hours after initiating lignocaine or adjusting dose<br>o Once patient has been on stable dose for 24hours monitoring can be reduce to every 6 hours<br>• <u>Toxicity/Signs and symptoms of Overdose</u>:<br>o Check for signs of toxicity at least once per shift and specifically record their absence in the nursing notes

The hard copy of this document may be out of date. To ensure you are reading the current version, check the policy and procedure site on the Alfred Health Intranet.

	○ If present, cease infusion, give supplementary oxygen, and immediately inform Acute Pain Service, or Palliative Care Service Dr.<br>• Daily 12 lead ECG or 3 lead for QTc assessment
**Drug Interactions**	Lignocaine is primarily metabolised by cytochrome P450 isoenzyme 1A2 in the liver, and to a lesser extent by 3A4 enzymes:<br>*Note: Lignocaine synonym is lidocaine. Use lidocaine to search in databases*<br>• **QT prolonging agents** – Azole antifungals, antipsychotics, some antidepressants e.g. citalopram, antiarrythmics, macrolides, methadone, quinolones & tyrosine kinase inhibitors especially nilotinib. See 2017 list of drugs that prolong QT and/or cause Torsades De Pointes or register for Credible Meds website for up to date information<br>• **Pro-arrhythmic agents** – antiarrhythmics esp. Class III potassium-channel blockers e.g. amiodarone & sotalol, macrolides e.g. clarithromycin, quinolones, azole antifungals and antipsychotics e.g. quetiapine[6,7]<br>• General Interactions – See Stockley online for specific interactions e.g. potent 3A4 inhibitors such as azoles<br>*This list only includes some of the common drugs and is by no means exhaustive. Contact your Clinical Pharmacist or call Medicines Information on 62002 for details relating to individual drugs.*
**Use in Pregnancy/Lactation**	See The Women's Hospital's online Pregnancy and Breastfeeding Medicines Guide

Key Related Documents

Alfred Health Drug Formulary Guideline

Neurology Resident Orientation Handbook 2016

ICU Management of Procedural Pain for Intubated Burn Patients Guideline

Lignocaine Infusion for Arrhythmia Guideline'

Peripheral Intravenous Cannulation & Ongoing Management Guideline: Adults

Niki T34 Subcutaneous Pump (Syringe Driver) Guideline

Lignocaine Infusion for Analgesic Rebound Headache/Migraine Guideline

Key Legislation, Acts & Standards

Charter of Human Rights and Responsibilities Act 2006 (Vic)[1]

References

1. (2014), *Cochrane Database Syst Rev, 10,* CD005622
2. Ashely C, Currie A. The Renal Drug Handbook. 3rd Edition. Radcliffe Publishing Ltd, U.K. 2009

[1] REMINDER: Charter of Human Rights and Responsibilities Act 2006 – All those involved in decisions based on this guideline have an obligation to ensure that all decisions and actions are compatible with relevant human rights.

The hard copy of this document may be out of date. To ensure you are reading the current version, check the policy and procedure site on the Alfred Health Intranet.

3. Jonsson A, Cassuto J, Hanson B. Inhibition og burn pain by intravenous lignocaine infusion. *Lancet* 1991; 338: 151-52.
4. Mao J, et al. Systemic lidocaine for neuropathic pain relief. Pain 2000; 87: 7-17
5. Peixoto R, Hawley P. Intravenous lidocaine for cancer pain without electrocardiographic monitoring: a retrospective review. J Palliat Med. 2015 Apr;18(4):373-7.
6. Schwartzman RJ et al. "Efficacy of 5-day continuous lignocaine infusion for the treatment of refractory complex regional pain syndrome" Pain Medicine 2009: 10(2); 401-412
7. Wasiak J, Cleland H Lidocaine for pain relief in burn injured patients(2007), *Cochrane Database Syst Rev, 18;(3),* CD005622 (R)
8. Wasiak J, Mahar PD, McGuinness SK, Spinks A, Danilla S, Cleland H, Tan HB Intravenous lidocaine for the treatment of background or procedural burn pain
9. Wasiak J, Spinks A, Costello V, Ferraro F, Paul E, Konstantatos A, Cleland H Adjuvant use of intravenous lidocaine for procedural burn pain relief: a randomized double-blind, placebo-controlled, cross-over trial (2011), *Burns, 37(6),* 951-7
10. Williams DR & Stark RJ. Intravenous lignocaine (Lidocaine) infusion for the treatment of chronic daily headache with substantial medication overuse. Cephalalgia 2003: 2; 963-971
11. Eipe N, Gupta S, Penning J. Intravenous lidocaine for acute pain: an evidence-based clinical update. BJA Education 2016;16(9):292-298
12. General resources also used in the preparation of this monograph include: *MIMS* [On-Line] (accessed 25/01/10); *Micromedex* [On-Line] (accessed 25/01/10); *Australian Medicines Handbook* [On-line (accessed 25/1/10); Burridge N (ed) *Australian Injectable Drugs Handbook*. 4th Edition. Melbourne: The Society of Hospital Pharmacists of Australia, 2008. *Pregnancy and Breastfeeding Medicines Guide.* Melbourne: The Royal Women's Hospital, 2010.

AUTHOR / CONTRIBUTORS

* denotes key contact

Name	Position	Service / Program
Kerry McLaughlin*	Consultant - APS	Surgical Services
Eliza Bell	Pain Liaison Nurse - APS	Surgical Services
Michelle Gold	Director, Palliative Care	Surgical Services
Cathy Corbett	Consultant, Palliative Care	Surgical Services
Alex Konstantatos	Head of Acute Pain Service, Consultant	Surgical Services
Thuy Bui	Analgesic Stewardship Pharmacist	Pharmacy
John Dalla	Palliative Care Nurse	Cancer & Medical Specialties
Mel Pacquola	Burns Liaison Nurse	Surgical Services
Heather Cleland	Director, Burns Unit	Surgical Services
Hana Menezes	Burns Nurse Practitioner Candidate	Surgical Services
Sarah Larwill	Clinical Services Director, Surgical Services	Surgical Services

Endorsed by:	Name/Title: Prof Michael Dooley – Director, Pharmacy	Date: 13 September 2017
Approved by:	Name/Title: Alfred Health Drug and Therapeutics Committee	Date: 13 September 2017
Minor amendment:	Approved by: Carmela Corallo – Formulary Manager	Date: 24 August 2020
Disclaimer: This procedure has been developed within the context of Alfred Health service delivery. Alfred Health shall not be responsible for the use of any information contained in this document by another organisation outside of Alfred Health.		

The hard copy of this document may be out of date. To ensure you are reading the current version, check the policy and procedure site on the Alfred Health Intranet.
