# Supplementary material for: Intravenous Lidocaine and Ketamine Infusions for Headache Disorders: A Retrospective Cohort Study
Source: Front Neurol. 2022 Mar 9;13:842082. doi: 10.3389/fneur.2022.842082 (PMC8959588; doi:10.3389/fneur.2022.842082)
Supplement: Supplementary file 2 [file Data_Sheet_2.PDF]

Title **KETAMINE FOR ANALGESIA AND SEDATION**

This medication guideline has been prepared to standardise the prescribing, administration and dispensing of this ketamine for analgesia and sedation for adult inpatients at Alfred Health. **For the use of ketamine in status epilepticus** refer to Status Epilepticus Management in Adults Guideline. **For paediatric sedation in ED**, Alfred Health has adopted the Royal Children's Hospital (RCH) clinical practice guideline *Ketamine use for procedural sedation*, endorsed by the Paediatric Improvement Collaborative. **The RCH guideline endorses use of ketamine in children from 3 months, but ketamine should not be used at Alfred Health for procedural sedation in children under two years of age without anaesthetic consultation.**
Additional information relating to this drug can be found in the references listed and by contacting the Alfred Medicines Information Service on 62002.

Areas Applicable: Operating Suites, ICU, ED, Trauma Unit Ward 5W Procedure Room, Ward 6W DUS Procedure Room, and general wards under Acute Pain Service, Palliative Care Unit or Neurology guidance.

Introduction
Ketamine is a phencyclidine derivative, mediating the majority of its actions via N-methyl-D-aspartate (NMDA) receptor antagonism. At low doses it produces analgesia and sedation, but at higher doses produces a dissociative anaesthetic state. Whilst airway tone, protective airway reflexes and ventilation are better maintained compared to anaesthetic states produced by other agents, they cannot be guaranteed. **Clinicians using ketamine boluses must have the necessary airway skills to obtain and maintain an airway and provide adequate ventilation when they are compromised**.

Tissue damage causing persistent nociception, or neuropathic pain states, may activate N-methyl-D-aspartate (NMDA) receptors in the spinal dorsal horn, producing the phenomenon of "wind-up", with the spinal hyperexcitability, allodynia and hyperalgesia (central sensitisation). NMDA antagonists, such as ketamine, can be used to attenuate this central sensitisation with consequent improved analgesia. Ketamine is often used in addition to opioids and has been shown to have an opioid-sparing effect.

**Description**	
**Drug Presentation**	• 200 mg (base)/2 mL (Ketalar® or alternative brand) – MUST BE DILUTED with compatible solution before administration<br>• 25mg wafer (Wafermine®) - for sublingual administration
**Prescribing & Administration Requirements /Restrictions for Medical Staff**	To reduce the risk of iatrogenic patient harm, the following restrictions apply:<br>• **Acute Pain Service (APS)**<br>○ Ketamine wafers are restricted to APS for analgesia during dressing changes in patients with burns (see Appendix 2)<br>• **Anaesthetic Staff**<br>○ **For Anaesthesia and Procedural Sedation:**<br>- Pre Part 1 Exam trainees are not to administer ketamine without direct supervision by a consultant<br>- Post Part 1 Exam trainees can administer ketamine, after discussion with a consultant<br>- Post Part 2 Exam trainees can administer ketamine without supervision or discussion with a consultant<br>○ **For Analgesia:**<br>- The decision to use ketamine as an analgesic adjunct can be made by the trainee who has completed 6 months of basic training, more junior trainee must discuss with a consultant.<br>- Bolus doses of ketamine prior to commencing an infusion should only be administered intra-operatively or in the Post-anaesthesia

The hard copy of this document may be out of date. To ensure you are reading the current version, check the policy and procedure site on the Alfred Health Intranet.

	Care Unit (PACU) by appropriately experienced staff<br>• **ED** refer to Appendix 1<br>• **ICU**<br>○ **For Anaesthesia and Procedural Sedation:**<br>-Only to be used under the supervision of, and after discussion with, an ICU consultant or senior registrar<br>○ **For Analgesia:**<br>-The decision to use ketamine as an analgesic adjunct infusion should be made after discussion with an ICU consultant or senior registrar<br>• **Neurology Unit**<br>○ Only used with the supervision of a Neurology Unit Consultant<br>○ Predominantly used as an intravenous infusion for treatment of migraine and neuropathic pain (*does not require APS consult*)<br>• **Palliative Care Unit**<br>○ Only used with the supervision of a Palliative Care Consultant<br>○ Predominantly used as a subcutaneous infusion<br>• **Trauma Unit in 5W Procedural Room**<br>○ Only used by credentialed **consultant** staff per Appendix 3
**Drug Storage/ Availability**	• S8 – stored in DD cupboard
**Actions (Pharmacology)**	• NMDA receptor antagonist<br>• In Australia, ketamine is a racemic mixture of the ‘r’ and ‘s’ enantiomers in a 1:1 ratio<br>• Rapid onset of dissociative anaesthesia, characterised by<br>○ Cataleptic state, often with eyes remaining open<br>○ Relative maintenance of pharyngeal/ laryngeal reflexes (subanaesthetic doses), but does not guarantee protection against aspiration in unfasted patients<br>○ Increased salivation, that can precipitate laryngospasm<br>○ Maintenance of minute ventilation (subanaesthetic doses)<br>○ Cardiovascular stimulation, with relative preservation of mean arterial pressure (MAP)<br>○ Normal or slightly enhanced skeletal muscle tone<br>○ Profound analgesia in sub-anaesthetic doses, and is opioid sparing Reduces ‘wind-up’ and may be useful in the treatment of neuropathic pain, and the prevention of chronic pain.<br>• Elimination half-life = 2 to 3 hours<br>• Metabolised to nor-Ketamine, which is an active metabolite with 20-30% activity of the parent compound<br>• 90% of ketamine excreted in urine (mostly as metabolites)<br>• Anaesthesia persists for only 5 to 10 minutes after an induction dose of ketamine; re-administration is necessary to maintain effective anaesthesia
**Indications**	**<u>1. INDUCTION OF ANAESTHESIA</u>**<br>• In ED, Operating suites, and ICU<br>• Only by clinicians with detailed knowledge of ketamine’s pharmacology and adequate experience with its clinical use. These staff must also have the requisite skills to manage a compromised airway and ventilation. Credentialing by each department/unit is defined in the Prescribing & Administration Requirements/Restrictions for Medical Staff section.<br>• Choice as induction agent for various operations, procedures, or

The hard copy of this document may be out of date. To ensure you are reading the current version, check the policy and procedure site on the Alfred Health Intranet.

	emergency intubation by appropriately credentialed clinician<br>• Usual dose as sole induction agent is 1-2mg/kg, and this may need to be significantly reduced in haemodynamically compromised patients, patients with reduced conscious states from any cause, and the elderly<br><br>**2. PROCEDURAL SEDATION/ANALGESIA**<br>• In ED, Operating suites, ICU, Trauma Unit Ward 5W Procedure Room, and 6W Procedure Room for Burns Dressings under Sedation (DUS)<br>• Only by clinicians with detailed knowledge of ketamine's pharmacology and adequate experience with its clinical use. These staff must also have the requisite skills to manage a compromised airway and ventilation. Credentialing by each department/unit is defined in the Prescribing & Administration Requirements/Restrictions for Medical Staff section.<br>• Choice as sedation agent for various procedures by appropriately credentialed clinician<br>• Usual dose to obtain conscious sedation is 0.5-1mg/kg, and this may need to be reduced in haemodynamically compromised patients, patients with reduced conscious states from any cause, and the elderly<br>• Patients must be adequately monitored to ensure adequate respiration and oxygenation<br>• As depth of sedation is increased, protective airway reflexes cannot be guaranteed, and unfasted patients are at risk of aspiration<br><br>**3. ANALGESIA**<br>• Intravenous, subcutaneous, and sublingual routes of administration<br>• Can be utilised on any general ward as follows:<br>○ Prescribed by **APS** as part of multi-modal analgesic regimen for any patient with severe pain, inadequately managed with opioids and conventional adjuvants.<br>○ Prescribed by **Palliative Care** physicians, as per the relevant guideline, in the treatment of severe chronic and/or cancer-related pain, especially those already on large doses of opioids.<br>○ Prescribed by the **Neurology Unit**, for the treatment of severe chronic migraines unresponsive to other agents, neuropathic pain with features of central sensitisation, Complex Regional Pain Syndrome (CRPS), and in patients in whom discontinuation of opiates is desirable.
**Contraindications**	**Absolute**<br>• Known hypersensitivity to ketamine products<br>• Previous severe adverse reactions, especially psychogenic<br><br>**Relative**<br>Use with caution if:<br>• Known or suspected difficult airway<br>• Unfasted and risk of aspiration<br>• Acute myocardial ischaemia<br>• Poorly controlled heart failure<br>• Uncontrolled hypertension<br>• Acute psychotic states and recent methamphetamine use<br>• Whilst not contra-indicated in patients with traumatic brain injury (TBI) and

The hard copy of this document may be out of date. To ensure you are reading the current version, check the policy and procedure site on the Alfred Health Intranet.

	raised intracranial pressure (ICP), the literature does not support its use as a sole agent, because it increases cerebral metabolic activity heterogeneously<br>• Whilst ketamine is probably safe to use in acute porphyrias, it is not the drug of choice, that being propofol.
**Precautions**	Use with caution:<br>• Conditions where hypertension is hazardous (e.g. cardiovascular disease, heart failure, uncontrolled hypertension, recent AMI, recent stroke, cerebral bleeding or mass). Problems unlikely at doses <0.3 mg/kg/hr by continuous infusion. Avoid bolus doses.<br>• Alcohol intoxication or a history of alcohol abuse<br>• Adverse psychogenic sequelae can be severe and occur in up to 15% of patients receiving anaesthetic doses. They are minimised by administering ketamine as an infusion and avoiding bolus doses. Due to the risk of these adverse psychological events, ketamine should be used in **caution in patients with psychiatric illness, acute psychotic states, or recent methamphetamine use,** whilst noting that it is recommended as third line treatment for the sedation of patients with acute behavioural disturbances in the ED, See Sedation for Acute Behavioural Disturbance in Emergency Department Guideline and Safer Care Victoria's Caring for people displaying acute behavioural disturbance**.**<br>• Glaucoma or elevated intraocular pressure, globe injuries.<br>• Hyperthyroidism (increased risk of hypertension, tachycardia)<br>• Do not use alone in surgery or diagnostic procedures of pharynx, larynx, or bronchial tree<br>• Consider dose reduction in patients with cirrhosis or hepatic impairment<br>• Potential abuse as a recreational drug
**Administration**	
**Dose Range**	**For IV Infusion Analgesia:**<br>• Usual Dosage Range: 0.1 - 0.3 mg/kg/hr (unless otherwise specified by the Consultant in charge)<br>• Usual Dose Range: 7 - 24 mg/hr<br>• Infusion Rate (4 mg/mL): 1.75 - 6 mL/hr<br><br>**IV Loading Dose prior to infusion***<br>Loading doses are *generally not recommended*, particularly on the general wards. A loading (bolus) dose should only be given to achieve adequate ketamine blood levels on initiation of infusion.<br>• Dose: 0.1 mg/kg over at least 1 minute to a **maximum** bolus dose of 10 mg IV. Consideration should be given to the addition of a bolus of midazolam 0.5 mg IV, particularly those predisposed to psychological manifestations.<br>• Dilute ketamine 200mg/2mL ampoule to 20mL with compatible fluid to produce a final concentration of 10mg/mL<br>**Bolus doses increase the risk of unpleasant psychological manifestations which include hallucinations and in some cases have caused susceptible patients to develop post-traumatic stress disorder. Bolus dosing should only be done by appropriately trained staff.*

The hard copy of this document may be out of date. To ensure you are reading the current version, check the policy and procedure site on the Alfred Health Intranet.

	**For Subcutaneous Infusion Analgesia:**<br>• **Acute Pain Service**<br>Same dosage range as above, and generally given via the PCA pump as a continuous infusion (at concentration as above for IV infusion)<br>• **Palliative Care Unit**<br>Generally start at doses of 100 mg/24 hours and increase incrementally up to doses of 500 mg/24 hours, via Niki T34 pump. Loading doses are not recommended in the Palliative Care setting. Note: For further advice on administration for continuous subcutaneous infusions liaise with Palliative care<br><br>**For migraine and neuropathic pain management by Neurology: Refer to IV Infusion Analgesia section.**<br><br>**For use in ED: Refer to Appendix 1**<br><br>**For Sedation + Analgesia in ICU:**<br>• 0.05– 0.4 mg/kg/hr<br>• Higher IV doses have been used for *sedation* and analgesia[4] but requires careful titration<br><br>**For Sublingual Analgesia (APS only): Refer to Appendix 2**
**Duration of Therapy**	• Need for ongoing therapy to be decided by Acute Pain Service, Palliative Care Unit, Neurology or ICU
**Titration Parameters**	• Titrate under direction of Acute Pain Service, Palliative Care Unit, ICU or Neurology for the approved indications<br>• Start at the lower end of the dose range to minimise side effects.<br>• Review at one hour – if pt comfortable leave at this rate, if patient has persistent pain consult relevant specialist team, i.e. Acute Pain Service, Palliative Care, Neurology or ICU<br>• Review at 6–12 hours – if comfortable leave at this rate, if not contact relevant specialist team and consider alternative techniques
**Intravenous Infusion**	**ICU and General Wards (use IV Ketamine powerplan)**<br>• Drug Dose: 200 mg (2 mL)<br>• Make up infusion **TO**: 50 mL in IV syringe<br>• Compatible Solutions: 0.9% sodium chloride, 5% glucose<br>• Final Concentration: 4 mg/mL<br>• 1mL/hr = 4 mg/hr<br>• Infusion pump: PCA pump (wards) or Alaris (ICU)<br>(In the event that a ketamine infusion is commenced by APS in ED, the PCA pump should be used unless the patient is to be transferred to ICU)<br><br>**Midazolam addition (APS Only):**<br>• Reduces the incidence of hallucinations in high risk patient groups ie. patients with neurotic traits or psychiatric illness<br>• **1mg Midazolam** added to the 200mg/50mL syringe<br><br>MUST have IV fluids running with the ketamine infusion at a minimum of 5mls per hour via an infusion pump, to prevent cannula occlusion and maintain drug

The hard copy of this document may be out of date. To ensure you are reading the current version, check the policy and procedure site on the Alfred Health Intranet.

	flow to the patient. The ketamine infusion line should be attached to the side arm of the IV maintenance line downstream of a non reflux valve **or** run with an existing infusion, if compatible.<br>*Note: Consultation with Acute Pain Service, Palliative Care or Neurology unit should occur prior to use on general wards*
**Ceasing infusion**	• This can usually be done over a few hours
**Incompatibilities**	• Barbiturates, diazepam, thiopentone
**Y-Site Compatibility**	• Morphine sulphate, oxycodone, midazolam, lignocaine (based on clinical practice). For other drug combinations please contact medicines information on ext 62002 or your clinical pharmacist.
**Subcutaneous Syringe Compatibility[7]**	• Midazolam, morphine sulphate. For other drug combinations please contact medicines information on ext 62002 or your clinical pharmacist.
**Practice Points**	
**Side Effects**	Side effects at analgesic doses are less common than at anaesthetic doses, but can be quite distressing to patients, especially hallucinations and related phenomena. Doses may need to be reduced or even ceased.<br><br>**Common**<br>• Increased salivary and tracheobronchial secretions<br>• Psychological manifestations varying in severity including (may occur during therapy and for up to 24 hours after therapy stopped):<br>• Pleasant dream-like states, floating sensations<br>• Emergence delirium<br>• Vivid imagery/hallucinations<br>• Confusion, insomnia, excitement, and irrational behaviour<br>• Treatment: Consider reducing rate of ketamine, or give low dose benzodiazepines (e.g. 0.5 mg IV/SC midazolam or sublingual 1 mg lorazepam using tablets) or haloperidol<br>*Note: These psychological effects occur to a lesser extent with the sub-anaesthetic analgesic doses and generally can be controlled by the treatment as recommended above.*<br><br>**Serious**<br>• Increased blood pressure (frequent)<br>• Tachycardia (frequent)<br>• Increased intraocular pressure<br>• Muscle hyperactivity (frequent), may resemble tonic/clonic seizures – more so at anaesthetic doses<br>• Respiratory depression (dose related or too rapid administration rate)<br>• Laryngospasm<br><br>**Other**<br>• Urinary tract toxicity/bladder dysfunction<br>• Rash, vomiting<br>• Local inflammation at subcutaneous infusion site

The hard copy of this document may be out of date. To ensure you are reading the current version, check the policy and procedure site on the Alfred Health Intranet.

<table>
<tr>
<td>Monitoring</td>
<td>
For ketamine infusions
<table>
<tr><th>FIRST <u>4</u> HOURS</th><th>NEXT <u>4</u> HOURS</th><th>ONGOING</th></tr>
<tr>
<td>1 Hourly
Heart Rate
Blood Pressure
Respiratory Rate
Sedation Score (0-3)
O2 Saturations
Pain Score (VNRS 0-10)
Functional Activity Score (FAS)</td>
<td>2 Hourly
Heart Rate
Blood Pressure
Respiratory Rate
Sedation Score (0-3)
O2 Saturations
Pain Score (VNRS 0-10)
Functional Activity Score (FAS)</td>
<td>4 Hourly thereafter
Heart Rate
Blood Pressure
Respiratory Rate
Sedation Score (0-3)
O2 Saturations
Pain Score (VNRS 0-10)
Functional Activity Score (FAS)</td>
</tr>
</table>

Monitor for hallucinations and vivid dreams. If patient experiences disturbing or distressing symptoms, cease ketamine and refer to medical team.
Monitor for signs of emergence delirium symptom. Monitor patient’s liver and renal function
Monitor SC injection site for local inflammation

</td>
</tr>
<tr>
<td>Drug Interactions</td>
<td>

Theophylline (decreases seizure threshold)
Use with caution if using with other medications that can increase the BP or heart rate. Monitor combinations closely.
Thyroxine (sustained rises in arterial blood pressure reported)
Potent cytochrome 3A4 inhibitors eg clarithromycin as ketamine is metabolised by this enzyme
Atracurium (ketamine may potentiate the neuromuscular blocking effects)
The use of ketamine with other central nervous system (CNS) depressants. Reduced doses of ketamine may be required with concurrent administration of other anxiolytics, sedatives and hypnotics
St. John's Wort (an increased risk of cardiovascular collapse and/or delayed emergence from anaesthesia). Cease St John’s Wort 5 days prior to ketamine administration.
Use with caution if using with drugs which prolong QTc interval (list available at https://www.crediblemeds.org/)

This list only includes some common drugs and is not exhaustive. Contact your Clinical Pharmacist or Medicines Information on 62002 for details relating to individual drugs
</td>
</tr>
<tr>
<td>Use in Pregnancy/Lactation</td>
<td>See The Women’s Pregnancy and Breastfeeding Medicines Guide</td>
</tr>
<tr>
<td>Other Considerations</td>
<td>Ketamine has been reported being used as a drug of abuse
For Patient Education Prior to Sublingual dose refer to Appendix 2</td>
</tr>
<tr>
<td>Nurse Practitioner/ Candidate Considerations</td>
<td>A Nurse Practitioner/candidate who works with the Acute Pain Service can prescribe a Ketamine infusion within the usual dose range parameters
Treatment of side effects extends to reduction or cessation of infusion only</td>
</tr>
</table>

The hard copy of this document may be out of date. To ensure you are reading the current version, check the policy and procedure site on the Alfred Health Intranet.

	• A patient who continues to have severe pain or intolerable side effects should be reviewed by an Acute Pain Service Consultant

APPENDICES

1. Administration in the Emergency Department
2. Ketamine Sublingual Administration For Dressing Change (INPATIENT USE ONLY)
3. Ketamine For Procedural Sedation In Ward 5 West Procedural Room

KEY RELATED DOCUMENTS

- Key aligned policy
  - Alfred Health Medication Safety Policy
  - Team Time Out Policy
- Key legislation, acts & standards:
  - Charter of Human Rights and Responsibilities Act 2006 (Vic)[1]
- Other relevant documents:
  - Acute Pain: Pharmacological and Non-Pharmacological Management in Adults Guideline
  - Referrals to Pain Management Services Guideline
  - Analgesia for Burns Dressing Changes Guideline
  - Patient Controlled Analgesia (PCA) Management Guideline
  - Recovery Room Management of Acute Pain in Adults Guideline
  - Sedation For Acute Behavioural Disturbance in the Emergency Department
  - ICU Sedation and Analgesic Titration Algorithm
  - Standardised Intravenous Bolus and Continuous Drug Infusions Table Guideline
  - Fasting Patient

REFERENCES

1. Owens, VF et al (2006) Ketamine: A Safe and Effective Agent for Painful Procedures in the Pediatric Burn Patient. Journal of Burn Care and Research. 27:211-216
2. Patterson, DR et al (2004) Pain management. Burns 30 : A10- A15
3. Reich, DL & Silvay G (1989) Ketamine: an update on the first twenty-five years of clinical experience. Canadian Journal of Anaesthesia 36: 186-197
4. Macintyre, PE & Ready, LB (2002) Acute Pain Management: a practical guide 2nd Edition, W.B.Saunders
5. Quibell R et al. Therapeutic Reviews: Ketamine. JPainSymMan 2011; 41:640-9.
6. Barr J et al. Clinical Practice Guidelines for the Management of Pain, Agitation, and Delirium in Adult Patients in the Intensive Care Unit. CCM 2013; 41:263-306.
7. Twycross R, Wilcock A & Howard P (2018). Palliative Care Formulary 6th Edition, Pharmaceutical Press.
8. Dickman A, Schneider J, Varga J. The Syringe Driver. Continuous subcutaneous infusions in palliative Care. 2nd Edn. Oxford University Press. Great Britain. 2005

[1] REMINDER: Charter of Human Rights and Responsibilities Act 2006 – All those involved in decisions based on this guideline have an obligation to ensure that all decisions and actions are compatible with relevant human rights.

The hard copy of this document may be out of date. To ensure you are reading the current version, check the policy and procedure site on the Alfred Health Intranet.

9. Tsai TH, Cha TL, Lin CM et al. Ketamine-associated bladder dysfunction. Int J Urol 2009:16:826-829
10. Zeiler FA, Sader N,et al. The cerebrovascular response to Ketamine:A systematic review of the animal and human literature. J Neurosurg Anesthesiol 2016; 28:123-140
11. Miller's Anesthesia, 9th Edition, 2020. Chapter 11, Cerebral Physiology and the Effects of Anaesthetic Drugs.
12. Yevgeny Filanovsky, MD;* Philip Miller, MD;† Jesse Kao, MDMyth: Ketamine should not be used as an induction agent for intubation in patients with head injury. CJEM 2010;12(2):154-7
13. Wafermine® product information http://www.ixbiopharma.com/wafermine
14. Jouguelet-Lacoste J, La Colla L, Schilling D and Chelly JE. The Use of Intravenous Infusion or Single Dose of Low-Dose Ketamine for Postoperative Analgesia: A Review of the Current Literature. Pain Medicine 2015;16:383-403.
15. Safe Care Victoria: Caring for people displaying acute behavioural disturbance.
16. ANZCA Committee for Pofessional Documents - endorsed by ANZCA A, GESA, RACS, CICM, RANZCR & RANZCP. PS09 - Guidelines on Sedation and/or Analgesia for Diagnostic and Interventional Medical, Dental or Surgical Procedures. ANZCA Professional Documents. 2014.

General resources also used in the preparation of this monograph include: *MIMS* [On-Line] (accessed 09/04/19); *Australian Medicines Handbook* [On-Line] (accessed 09/04/2019); Burridge N (ed) *Australian Injectable Drugs Handbook* [On-Line] (accessed 09/04/19); *The Women's Pregnancy and Breastfeeding Medicines Guide* [On-Line] (accessed 09/04/19].

KEYWORDS

Ketamine

AUTHOR / CONTRIBUTORS

* denotes key contact

Name	Position	Service / Program
Thuy Bui*	Lead Analgesic Stewardship Pharmacist	Pharmacy
Olivia Pellicano	ICU Pharmacist	Pharmacy
Cristina Roman	Emergency Pharmacist	Pharmacy
Trang Nguyen	Senior Pharmacist – Sandringham	Pharmacy
Judit Orosz	Intensive Care Physician	ICU
Catherine Bell	Clinical Nurse Consultant	ICU
Robert Melvin	Deputy Director	Emergency
Glenn Downey*	Deputy Director, Head of Clinical Services	Anaesthesiology and Perioperative Medicine
Alex Konstantatos	Director of Pain Services	Pain Services
Eliza Palfery	Acute Pain Nurse	Pain Services
Michelle Gold	Director, Palliative Care Service	Palliative Care
Julian Ellis	Neurology Pharmacist	Pharmacy
Elspeth Hutton	Head of Peripheral Neurology, Pain and Headache	Neurology
Simon Hendel	Anaesthesiologist, Head of Trauma Anaesthesia	Anaesthesiology and Perioperative Medicine
Mark Fitzgerald	Director of Trauma	Trauma Services

Appendix 1 – Administration in the EMERGENCY DEPARTMENT

Only to be used under the supervision of, and after discussion with, an Emergency Physician, or ED Fellow/Senior Registrar if at night.

- Pre-Primary Exam trainees are not to administer ketamine without direct supervision by an Emergency Physician
- Post-primary Exam trainees can administer ketamine after discussion with an Emergency Physician or ED Fellow.

In addition to above, trainees administering ketamine must have completed their LEX module for Procedural Sedation.

Administration	
**Dosing#**	**1. Intractable pain**<br>• Where maximal doses of narcotics have been reached, or if patient instability necessitates use<br>• Dose*: IV 0.1-0.5mg/kg over at least 1 minute#<br>• Consideration should be given to the addition of a bolus of midazolam 0.5 mg IV<br>• Note: Early notification of the Acute Pain Service is required if difficult analgesic requirements are anticipated or if more than two bolus doses are needed.<br>*A maximum of two bolus doses may be used in the ED by experienced medical staff, prior to consultation with the Acute Pain Service.<br><br>**2. Procedural Sedation**<br>• When indicated due to patient instability, fasting status or where other agents are less appropriate and procedure cannot be delayed<br>• Dose: 0.5-1mg/kg plus a further 0.5mg/kg if required. Give by slow IV push.<br>• Consider giving midazolam prior to ordering procedure to limit psychological manifestations, particularly in susceptible patients.<br><br>**3. Induction of Anaesthesia**<br>• When indicated due to patient instability, or where considered to be the safest agent (e.g. airway issues, bronchospasm). May be used as a single agent or in combination (e.g. with benzodiazepines)<br>• Dose: IV 1-2mg/kg.# Give by slow IV push.<br>• Use caution in hypertensive patients<br>***Note-*** *Prior agents used for analgesia, particularly high dose opioids, may necessitate a reduction in the bolus dose to be given. Without dose reduction, maintenance of normal airway reflexes and ventilation may be compromised.*<br>#Dilute ketamine 200mg/2mL ampoule to 20mL with compatible fluid to produce a final concentration of 10mg/mL

The hard copy of this document may be out of date. To ensure you are reading the current version, check the policy and procedure site on the Alfred Health Intranet.

Appendix 2 - Ketamine Sublingual Administration For Burns Dressing Change (INPATIENT USE ONLY)

Description	
**Presentation – Sublingual dose**	• 25mg wafer (Wafermine®)<br>• S8 – stored in DD cupboard on ward

Administration	
**Dosing**	**To be used in ICU and General wards following consultation with Acute Pain Service**<br>• **LOADING DOSE:** Sublingual ketamine (25 mg) administered 10 minutes prior to commencement of dressing change (I.e. before dressing removal).<br>• **BOLUS DOSES:** The patient can receive extra doses of sublingual ketamine during the dressing change as they require (5 minutely up to 3 times PRN). The boluses doses are only to be **patient initiated** (at the patient's request). The maximum total dose that the patient receives is at the acute pain services discretion and will vary from patient to patient.

Counselling	
**Patient Education Prior to Sublingual dose**	• Advise patient that the dose is to be placed under the tongue and the patient should try to not swallow the medication for as long as possible<br>• Prior to commencement of sublingual ketamine, the patient should be educated on the medication and the possible side effects such as:<br>• Bitter taste<br>• Can give a "warm" or "weird" effect,<br>• Hallucinations are possible but unlikely<br>• Older patients may not like dysphoric effect<br>• Short acting<br>• **Do not repeat dose** if patient disliked the effects
**Patient Education on Discharge**	Advise patient that the effect of ketamine can last for up to 24 hours after the last dose.<br>• Patient to remain in bed for 60mins after final ketamine dose<br>• They should be cautioned about driving or engaging in hazardous activities etc during this time period<br>• Counselled that emergent psychological reactions may occur

The hard copy of this document may be out of date. To ensure you are reading the current version, check the policy and procedure site on the Alfred Health Intranet.

Appendix 3 – Ketamine For Procedural Sedation in Ward 5 West Procedural Room

**** Only credentialed CONSULTANT Trauma medical staff are authorised to administer ketamine sedation on 5 West ****

In general, to be *credentialed* to administer IV ketamine sedation on 5 West, practitioners must be:

- An emergency physician (FACEM) and be credentialed to use ketamine sedation in ED, or
- A specialist anaesthetist (FANZCA), or
- A trauma consultant (FACEM, FANZCA, FCICM, FRACS or equivalent) and,
  - Have had previous experience in the use of ketamine sedation, or
  - Competed training in the use of ketamine sedation (including observing ketamine sedation and performing ketamine sedation under supervision)

Indications

- Procedures requiring IV sedation (e.g. insertion of intercostal catheters)

Staffing

- In accordance with joint ANZCA/ACEM PS09 guideline,[16] a minimum of three staff are required:
  - 1 credentialed consultant doctor (the sedationist),
  - A second doctor (the proceduralist), and
  - A third assistant (nursing or medical staff)

Consent / Assessment

- Consent should be obtained for procedural sedation either from the patient or, if not possible, from the appropriate medical decision maker
- Pre-sedation assessment should include, as a minimum:
  - *Airway* assessment - any suspected difficult airway should not have procedural sedation without consultation with anaesthesia
  - A *fasting* assessment – patients should be fasted in accordance with Alfred Health fasting guidelines
  - A *pre-procedure* assessment including allergies, past medical history, and current medications
- A "time-out" should occur prior to the procedure in accordance with Alfred Health guidelines

Monitoring

- Patients should be monitored with a minimum of[16]:
  - Pulse oximeter and
  - Non-invasive blood pressure
- All patients should be provided with supplemental oxygen while sedation occurs
- Equipment for monitoring end-tidal carbon dioxide should be available on the ward, as should emergency drugs, ECG monitoring and equipment for endotracheal intubation
- Suction equipment should be available and checked prior to commencing each sedation case

Prescribing / Dosage

- Dilute ketamine 200mg/2mL ampoule to 20mL with sodium chloride 0.9% to produce a final concentration of 10mg/mL
- IV access patency should be confirmed prior to commencing injection of ketamine
- Sedation with ketamine should commence with slow IV injection, and the initial dose should not exceed **0.5mg/kg**
- **The total dose of ketamine for sedation should not exceed 1mg/kg**

Recovery

- Patient should remain in the procedure room (or other appropriately monitored environment), with 1:1 nursing and minimum monitoring (pulse oximetry and BP) until conscious or for a minimum of 30 minutes after the procedure.

The hard copy of this document may be out of date. To ensure you are reading the current version, check the policy and procedure site on the Alfred Health Intranet.
